# A Systematic Review of the Needs of Children and Young People of a Parent Diagnosed With Young‐Onset‐Dementia: Informing a Nursing Intervention

**DOI:** 10.1111/jan.70205

**Published:** 2025-09-08

**Authors:** T. Sobers, E. Wolverson, H. Gardner, P. Joddrell, M. Walpert, A. Pepper, K. Harrison Dening

**Affiliations:** ^1^ Dementia UK London UK; ^2^ Geller Institute of Ageing and Memory (GIAM) University of West London London UK; ^3^ School of Medicine and Population Health (ScHARR) University of Sheffield Sheffield UK; ^4^ Health & Life Sciences, De Montfort University Leicester UK

**Keywords:** admiral nurse, children, needs, nurse, systematic review, young onset dementia, young people

## Abstract

**Aim:**

The aim of this study was to understand the needs of children and young people of a parent with young‐onset dementia, to inform the development of a nursing model.

**Background:**

Children and young people of a parent diagnosed with young onset dementia have a range of needs that are subject to change and aligned to their stage of development and growth.

**Design:**

Systematic review.

**Data Sources:**

Searches were conducted in PsycInfo (1806–Jan 2025), Medline (1996–Jan 2025) and CINAHL (1961–Jan 2025); search terms were developed in consultation with an academic librarian.

**Review Methods:**

The Preferred Reporting Items for Systematic reviews and Meta‐Analyses was used to assess the trustworthiness and applicability of the findings and the Mixed Methods Assessment Tool to assess quality. The review protocol was registered on PROSPERO (CRD42024534104). Needs identified from the literature were matched with the activities and interventions of a specialist nursing model.

**Results:**

Searches yielded 223 records of which 17 met the inclusion/exclusion criteria, the majority of which used qualitative methods (*N* = 16). A thematic synthesis approach was used to analyse data to reveal four emergent themes: (1) finding a way, (2) social connection and peer support, (3) preserving childhood and adolescence and (4) practical support, including the needs relating to education. Identified needs: knowledge and information, emotional support, consistency in education and development, maintaining social connections, physical and psychological well‐being, and grief and loss were mapped against a specialist nurse role.

**Conclusion:**

Children and young people with a parent diagnosed with young‐onset dementia face unique challenges compared to older carers. Despite growing awareness of their needs, this population is often overlooked in national dementia strategies. Developing a specialist nurse role is a positive step, but broader systemic support is essential to safeguard their well‐being and future opportunities.

**Reporting Method:**

This study adheres to the PRISMA reporting guidelines.

**Patient or Public Contribution:**

A bespoke Research Advisory Group, consisting of people with young onset dementia, young family carers, clinicians and academics, guided the review.

**Summary**

- Why is this research or review needed?
  ○ The needs of children and young people of a parent diagnosed with young onset dementia (YOD) have received international research attention for over 10 years.
  ○ Understanding and meeting the distinct needs of children and young people affected by YOD is crucial in supporting them to cope.
- What are the key findings?
  ○ Many children and young people experience pressures, issues, and needs common to adult carers of a person with dementia.
  ○ Despite the needs of children and young people of a parent diagnosed with YOD being known, few countries have responded with a service offer.
  ○ The identified needs in the literature can inform the intervention of a specialist nursing model.
- How should the findings be used to influence policy/practice/research/education?
  ○ The needs of children and young adults need to be included in national dementia policy.
  ○ This review contributes to the development of an innovative nursing model which aims to meet the identified needs of children and young people of a parent with YOD.

## 1 Introduction

It is estimated that there are 1 million people living with dementia in the United Kingdom (Wittenberg et al. 2019, 2020). Of those, approximately 70,800 have ‘young onset dementia’ (Carter 2022), a term used to describe the development of symptoms before the age of 65 years (Rossor et al. 2010). A range of causes for young onset dementia (YOD) have been reported, with the most common being Alzheimer's disease (AD) and frontotemporal dementia (FTD) (Loi et al. 2023).

Many individuals diagnosed with YOD are still working, caring for children and older relatives, and have a spouse (Knight and Pepper 2024). Therefore, there is a substantial familial impact associated with the condition, as families coping with YOD face unique unmet needs and complex challenges, due to their age and symptom presentation. Young onset dementia often presents with neuropsychiatric symptoms and can lead to various socio‐emotional challenges. These may include psychosis, behavioural and personality changes, lack or loss of empathy, disinhibition, irritability, compulsive behaviours, apathy, aggression, and difficulties with planning, judgement and communication (Draper and Withall 2016; Griffin et al. 2016; Rossor et al. 2010). Lengthy delays in receiving a diagnosis are often reported, with some individuals experiencing delays of up to 5 years (Kvello‐Alme et al. 2021). Misdiagnosis is common, with symptoms attributed to marital problems, depression, menopause, occupational stress or alcohol issues (Draper and Withall 2016; O'Malley et al. 2021). There is also a lack of recognition of the needs of people with YOD within healthcare. Services, formal support and resources are often inadequate, non‐specialist and not age‐appropriate (O'Malley et al. 2022; Wiggins et al. 2023). Where there are resources, they are far fewer than those available for late‐onset dementia, leaving families feeling isolated and stigmatised.

For families and carers, YOD can have a substantial negative psychosocial impact, including psychological distress, high levels of carer burden and low quality of life (Chirico et al. 2022; Wiggins et al. 2023). There is growing recognition and concern for how children and young people within these families might be affected as they transition from childhood, through adolescence to adulthood. Children and young people of a parent diagnosed with YOD may take on the role of carer, and often remain hidden unrecognised or unsupported by mainstream services (Sprung and Laing 2017). Despite their similarities in experiences and need for support, as with other carers, children and young people have additional requirements that are both contemporary and subject to change, aligned to their stage of development and growth. Therefore, understanding and meeting the distinct needs of children and young people affected by YOD is crucial in supporting them to cope.

There is a growing body of research that has explored the needs of children and young people of a parent with YOD (Poole and Patterson 2020; Grundberg et al. 2021) with recommendations suggesting that the core clinical functions of a YOD service should reflect family circumstances and include support and advice for family members, informal carers and dependent children (Young Dementia Network 2021; Royal College of Psychiatrists 2018). As problems occur across the course of YOD, from the often‐lengthy time required to obtain a diagnosis, to receiving post‐diagnostic support and palliative and end‐of‐life care, addressing the needs of children and young people should be a priority for service providers. The needs of families affected by YOD are a strategic and research priority for Dementia UK, the charity that supports the development and growth of Admiral Nursing, and specifically the needs of children and young people of a parent diagnosed with YOD.

As a specialist nurse in dementia care in the UK, Admiral Nurses support people living with dementia and their families, including those with YOD (Gardner and Pepper 2023; Knight and Pepper 2024). They provide case management and work in a range of care settings such as clinics, communities, and hospitals, adopting a family‐centred approach to support families across the illness trajectory (Harrison Dening et al. 2017). This paper details a systematic review of the literature on the needs of children and young people of a parent with YOD, which has been used to further inform the development of an innovative model of Consultant Admiral Nurse for Children and Young People to provide age‐appropriate care and services where needs identified in the systematic review have been used to develop specific aspects of the role.

### 1.1 Objectives

This systematic review aimed to address the following question of the literature:

What are the psychosocial needs of children and young people (0–25 years) with a parent diagnosed with young onset dementia.

From identifying these needs, we then explored how the role and function of an Admiral Nurse could be bespoke to meet these identified needs.

## 2 Methods

The protocol for this systematic review was co‐created with a research advisory group (RAG) comprised of people living with YOD, young people with a parent diagnosed with YOD, Dementia UK Business Development team, Admiral Nurses (specialising in YOD) and researchers active in this field. The review questions arose through regular consultation with the RAG, with members helping to develop the search questions and strategy. The protocol was registered on PROSPERO (CRD42024534104).

### 2.1 Search Strategy

The search terms were developed using Medical Search Term headings (MeSH), and database selection was made in consultation with an academic librarian and following a review of similar published literature. It was felt that most databases index articles under “dementia” or “Alzheimer's disease” and therefore a list of subtypes was not included. Three databases, PsycINFO (1806–present), MEDLINE, and CINAHL, were selected for their relevance to psychological, biomedical, and allied health literature with search alerts set and reviewed until the submission of the article. This combination ensured comprehensive coverage of research, including qualitative, quantitative, and mixed methods, on the psychosocial and clinical aspects of young‐onset dementia affecting children and families, while maintaining a focused and manageable scope for the review. Mixed methods reviews are reputed to gain unique insights related to complex health care issues (Stern et al. 2020).

MeSH search terms included:

(“working age” OR “young* onset” OR “earl* onset” OR “under 65” OR “young people”)

AND

(dement* OR alzheimer*)

AND

(Child* OR “young person” OR “young people” OR “young carer”)

AND

(need* OR “psychosocial*” OR “support*” OR “unmet need*” OR “psychological”)

In addition, reference lists from reviewed papers were used to identify any additional and relevant studies. Articles were exported into and managed using the web app of Papers version 5 (ReadCube); Rayyan, the web‐based review management software, was used to remove duplicates and facilitate title, abstract, and full‐text screening.

### 2.2 Eligibility Criteria

Primary literature reporting on the psychosocial needs of children and young people with a parent with YOD of any subtype or cause, where symptom onset occurred before the age of 65 years, was included or excluded according to the following criteria (See Table 1).

**TABLE 1:** Inclusion and exclusion criteria.

	Justification
Inclusion criteria	
Studies of children and young people where parental diagnosis of young‐onset dementia occurred before they (children and young people) were aged 25 years	There is considerable debate on defining children and young people. Previous reviews have included individuals up to 24 years old (Wang and Brooke 2020) and others up to 35 years old (Chirico et al. 2022). After discussing with our RAG, we agreed on 25 years, embracing the UK national average age for leaving home (Office for National Statistics (ONS) 2021)
Studies that had an explicit aim to look at accounts and experiences of children and young people, which could include retrospective accounts from the now ‘adult child’	Direct accounts and experiences of children and young people were the focus of the study. Retrospective accounts were included as the RAG considered they could provide useful long‐term insights and reflections
Studies that considered mixed populations (e.g., children and spouse without dementia), were included where data relating to children and young people was clearly identifiable and could be extrapolated	
Studies reporting results of primary research using either qualitative, quantitative or mixed methods	
Literature was published in English	No resources for translation
Exclusion criteria	
Secondary sources of literature, including systematic reviews	Primary research studies exploring the experiences and needs of children and young people were the focus of the review

### 2.3 Study Screening and Selection

**FIGURE 1:**
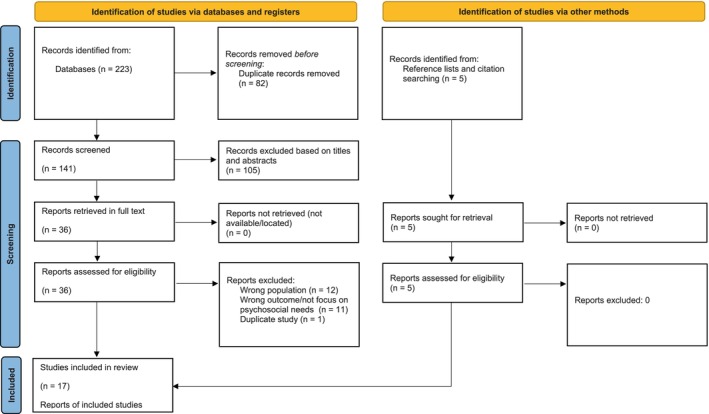
PRISMA study flow.

Initially, one reviewer (TS) screened titles and abstracts, excluding those not meeting the inclusion criteria (See PRISMA, Figure 1). Full‐text articles were then obtained for closer investigation. Reference lists of relevant literature reviews were scanned for studies for inclusion. Two reviewers (TS and EW) independently screened all identified studies, resolving any discrepancies about eligibility through discussion.

### 2.4 Quality Assessment

All studies which met the initial inclusion criteria were subject to quality assessment. The Mixed Methods Appraisal Tool (MMAT) (Hong et al. 2018) was used to assess the quality of each study. The MMAT tool is validated and reliable in appraising the quality of different study designs and enables an efficient appraisal process. Two authors (TS and EW) reviewed independently, and disagreements were resolved by a third author (KHD). Low quality studies were not excluded from the review, but quality assessment was considered in the analysis stage of the review. The decision not to exclude based on quality rating was made given the limited literature in the area and a desire to provide an overview of the current research landscape.

### 2.5 Data Extraction and Synthesis

Data extraction was performed by one member of the review team (TS) with observation/accuracy checks by another review team member (EW). Data were tabulated from all eligible studies (authors, year of publication, country where research was conducted, study aim, description of the study sample, study design, summary of findings related to psychosocial needs and conclusion). A thematic synthesis approach was used to analyse primary qualitative research data (Thomas and Harden 2008). The process involved line‐by‐line coding, developing descriptive themes, and generating analytical themes. Codes were created inductively to capture the findings' meaning and content. Initial codes were reviewed until no new codes emerged, ensuring consistency and accurate interpretation. Similarities and differences in codes were identified, leading to the formation of seven descriptive themes. These themes were then refined through discussion with all authors, resulting in four analytical themes that met the research aims.

Member checking (Birt et al. 2016) was then conducted with the RAG for discussion and comment. Feedback was incorporated into the refinement of the analytical themes and further development of the results and the discussion.

## 3 Results

Searches yielded 223 records (see PRISMA Figure 1). After duplicates (*N* = 82) were removed, 141 were screened for eligibility by the first author based on titles and abstracts. Of these, 105 were excluded, and the full text of 36 of the remaining articles was obtained. Hand searching the reference lists of these full‐text articles yielded an additional five papers that were included and obtained in full text. A total of 43 articles were assessed for eligibility, and 24 were excluded. A total of 17 studies were included in the review.

### 3.1 Study Characteristics (See Table 2)

**TABLE 2:** Study characteristics.

Study and location	Aim	Participants	Methods	Findings (in relation to children and young people)	Conclusion
Allen et al. (2009) UK	To explore the impact of young people's well‐being of having a parent with younger onset dementia	Children 12 (m = 5, f = 7) (13–24 years)	Qualitative Interviews Grounded theory	One overarching theme; 1 day at a time in response to the perception of severe threats in the future	Similarity to stress‐process models of caregiving with distinctive features arising from the interaction of YOD and the developmental stage of the child
Aslett et al. (2019) UK	To explore the experience of young adults having a parent with YOD	Children 5 (m = 2, f = 3) (23–36 years)	Qualitative Interviews Interpretative phenomenology	Five themes; relationship changes, shifts in roles and responsibilities, support for the non‐affected parent, support for self and the impact of living with their own potential risk of dementia	Children with a parent with YOD have specific needs which service providers should consider in the wider context of YOD care
Gelman and Greer (2011) US	To illustrate the impact the psychosocial impact of YOD on children	Children 3 (m = 1, f = 2) Mother (*n* = 1) (12–40 years)	Qualitative Case review/study	The review illustrates the psychosocial impact on children using the case of a father with YOD and his two children	There are limited resources and services currently available to support children with a parent with YOD. There is a need for age‐appropriate interventions to meet the unique needs of child caregivers
Gelman and Rhames (2018) US	Report on the experiences and needs of children of a parent with YOD and impact of scholastic, physical, emotional and social development	Children *n* = 8 (m = 3, f = 5) (15–20 years) Mothers (*n* = 4) (43–51 years)	Qualitative Semi‐structured interviews Thematic analysis	Children report disruption to developmental trajectory, emotional and psychological development, familial and broader social relationships, and financial instability	Learning can be used to educate parents, teachers, and other service providers of the critical needs of children and inform the development of targeted services and policies for children of a parent with YOD
Gelman and Rhames (2020) US	To examine the effect of YOD on (mother's parenting capacity and) children's experiences	Children *n* = 8 (m = 3, f = 5) (15–20 years) Mothers (*n* = 4) (43–51 years)	Qualitative Semi‐structured interviews Thematic analysis	Children assume roles of carers and earners, and at times reluctant decision‐making though reluctant. Responsibilities overwhelming at times, though reluctant to burden parent with their feelings and experience. Divergent experiences of YOD on well parent and on children	There is a dearth of information, resources, and services tailored to the needs of children of a parent with YOD
Groennestad and Malmedal (2022) Norway	To explore the experiences and perceptions of young adult children of a parent with YOD with focus on personal lives, family and social relationships in a Norwegian context	Children 10 (f = 10) (19–30 years)	Qualitative Semi‐structured interviews Thematic analysis	Six themes; upon discovering dementia, keeping the family together, others do not understand, sense of relief, need for support and apprehension for the future. Participants struggled with managing new responsibilities whilst preserving their own lives. They experienced a lack of understanding and support from others	Having a parent with YOD is a challenging life situation. Children the need a person‐ and family‐oriented approach
Hutchinson, Roberts, Daly, et al. (2016) Australia	To explore the lived experience of young people living with a parent with YOD from the perspective of the social model of disability	Children 12 (m = 1, f = 11) (10–33 years)	Qualitative Semi‐structured interviews Thematic analysis	Four themes identified: the emotional toll of caring, keeping the family together, grief and loss and psychological distress	A ‘whole family’ approach is proposed, where the needs of young people and their parents are respected and responded to age appropriately
Hutchinson, Roberts, Kurrle, and Daly (2016) Australia	To explore the lived experiences of young people having a parent with YOD To explore influencing factors that could enable young people to be included and supported within their community	Children 12 (m = 1, f = 11) (10–33 years)	Qualitative Semi‐structured interviews Focus group Thematic analysis	Three themes; invisibility; connectivity; empowerment. Identifying the basic human right to receive the age‐appropriate support	A shift by society is required to develop inclusive cross‐sectorial cooperation linking service providers across youth and dementia sectors. This requires working in partnership with the service users responding to the identified needs of children of a parent with YOD
Johannessen et al. (2015) Norway	To investigate and interpret the stories of children of persons with YOD	Children 14 (m = 5, f = 9) (18–30 years)	Qualitative Interviews Metaphor analysis	Four core metaphors abstracted: my parent is sliding away; emotional chaos; becoming a parent to my parent; and a battle	Growing up with a parent with YOD impacts on their personal development. Children have unmet needs for support, tailored information and follow‐up are required
Johannessen et al. (2016) Norway	To explore how adult children experienced the influence of their parents' dementia on their own development during adolescence	Children 14 (m = 5, f = 9) (18–30 years)	Qualitative Interviews 2 times, 1 year apart Grounded theory	Children used different instrumental, cognitive, and emotional coping strategies, from which two themes emerged; 1 detachment; subthemes were moving apart, greater personal distance, and calmer emotional reactions. 2 Resilience, which related to combinations of the coping strategies employed	Informants experienced a better life situation and less emotional stress over time as their parent's dementia progressed, developing better coping capacities and greater resilience. Vital throughout was the need for social support
Lord (2009) UK	To explore the continuing psychological impact of having a father with YOD *DClinPsy thesis and follow up study to Allen et al. (* *2009* *) where some participants were re‐interviewed*	Children 7 (m = 3, f = 4) (17–28 years)	Qualitative Semi‐structured interviews Grounded theory	Children of a parent with YOD experience multiple losses and grief over its course; with some differences between the child and spouses of the person with YOD	
Masterson‐Algar et al. (2023) UK	To explore the factors that play a role in the success (or failure) of relevant services/organisations and research to identify (and support) young dementia carers	Participants 19 professionals & researchers (*n* = 14), people with lived experience (*n* = 5), of which (*n* = 1) was a child *Age not reported*	Qualitative Semi‐structured interviews (*n* = 17) Participatory online workshop (*n* = 15) Thematic analysis	Need for a broad and holistic approach to the identification and support of children of a parent with YOD. Support should be accessible, relatable, and tailored to their needs. Study makes six recommendations for research and practice, including evaluation of the dedicated Admiral Nurse model for children of YOD	Children of a parent with YOD are currently unsupported and unidentified by services and wider society
Millenaar et al. (2014) Netherlands	To explore the experiences of children living with a young parent with dementia with a specific focus on the children's needs	Children 14 (m = 6, f = 8) (15–27 years)	Qualitative Semi‐structured interviews Content analysis	Three themes; the impact of dementia on daily life, different ways of coping, and children's need for care and support	In addition to practical information, more accessible and specific information about the diagnosis and the course of YOD is needed to provide a better understanding of the disease for the children also the need for a personal, family‐centred approach
Nichols et al. (2013) US & Canada	To learn more about the needs and experiences of young carers for patients of frontotemporal dementia in order to create a relevant support website for young caregivers to dementia patients	Children 14 (m = 4, f = 10) (11–18 years)	Qualitative Focus groups Thematic analysis	Seven overlapping themes: emotional impact of living with a parent with FTD, caregiving, coping, symptoms, diagnosis, relationships, and support	Young carers experienced caring for a parent with YOD as positive overall, but identified needs in overcoming stigma and balancing childhood and adolescent development within this context
Sikes and Hall (2018a) UK	To explore impact of parental YOD on educational careers	Children 24 (m = NK, f = NK) (6–31 years) (*n* = 4 over age 25)	Qualitative Interviews over 18 months Grounded theory	Living with a parent with YOD, makes negotiating the education system extremely hard. Exacerbations experienced by YOD distinctive characteristics, lack of cure and anticipatory, pre‐death grief. Lack of public awareness and understanding, dedicated resources and support services, contribute to feelings of isolation and of being ignored	Children and young people require support if they are to fulfil their educational potential and enjoy the social opportunities. Schools, colleges and universities are uniquely placed to provide such support
Sikes and Hall (2018b) UK	To explore the experiences of children and young people who have a parent with dementia	Children 19 (m = 3, f = 16) (8–31 years)	Qualitative Interviews (auto/biographical) Thematic analysis	Literature proposes that people with dementia are ‘still’ the same person they were prior to the onset of their condition, however, for children this is not helpful. Expectations that they will behave and feel towards that parent as they did before are a source of distress	Support for children needs to acknowledge that their parent may well be different to the Mum or Dad they previously ‘knew’
Svanberg et al. (2010) UK	To discover the experiences of the children of people with YOD and the explore the impact of the diagnosis	Children 12 (m = 6, f = 6) (11–17 years)	Mixed Interviews Measures: MFQ, ZBI‐Short, & RS Grounded theory	Four categories identified: discovering dementia; developing a new relationship; learning to live with it and going through it together. A three‐stage process model of adapting to dementia is proposed; moving through grief to emotional detachment and increased maturity	Developing a whole family approach for young carers can reduce burden

Abbreviations: f, female; FTD, frontotemporal dementia; m, male; MFQ, Mood and Feelings Questionnaire; RS, Resilience Scale; YOD, young onset dementia; ZBI‐Short, Zarit Burden Interview (Short).

The first study reviewed was conducted in 2009 and the most recent was in 2023. The number of studies during those years peaked between 2016 and 2018. Eleven studies were conducted in Europe (seven of which in the UK), four in North America and Canada, and two in Australia. The majority of studies (*N* = 16) were qualitative in design, including grounded theory, interpretive phenomenology, case review, semi‐structured interviews, focus groups, participatory workshops, and one using mixed methods incorporating quantitative outcome measures with grounded theory (Svanberg et al. 2010). Eleven studies aimed to report on the overall experiences of children and young people of having a parent with a diagnosis of YOD. The second most significant aim was to understand the psychosocial impact, such as on education, relationships, and development and needs that arise.

Sample sizes ranged from 1 to 24 of children and young people of a parent diagnosed with YOD; the total number of individual participants was 156, as some studies reported on different analyses of the same sample (Gelman and Rhames 2018, 2020; Hutchinson, Roberts, Daly, et al. 2016; Hutchinson, Roberts, Kurrle, and Daly 2016; Johannessen et al. 2015, 2016; Sikes and Hall 2018a, 2018b). The age range of children and young people was reported between 10 and 36 years (note: as per inclusion and exclusion criteria, where the age range exceeded the inclusion criteria of 25‐year, the study was included if data could be extrapolated that related to a specific age) the majority being female (*N* = 91), though two studies did not report the gender of the children or young people (Masterson‐Algar et al. 2023; Sikes and Hall 2018a).

### 3.2 Quality of Studies

Quality assessment using the MMAT was conducted for the 17 included studies. MMAT standards for data collection methods, analysis, interpretation of findings, and coherence across these domains were satisfactorily met for most studies. However, four studies demonstrated limitations in the areas of analysis and reporting of results/data (Gelman and Greer 2011; Johannessen et al. 2016; Sikes and Hall 2018b; Svanberg et al. 2010) (File S1 available). In line with MMAT recommendations, overall quality scores were not calculated (Hong et al. 2018).

#### 3.2.1 Themes

The following four emergent themes, present throughout the studies, highlight the psychosocial needs of children and young people of a parent diagnosed with YOD: (1) Finding a way, (2) Social connection and peer support, (3) Preserving childhood and adolescence, and (4) Practical support, including the needs relating to and the role of education. Findings from the one study that employed mixed methods (Svanberg et al. 2010) have been incorporated into the themes.

##### 3.2.1.1 Theme 1: Finding a Way

The psychological and emotional impact of living with a parent with YOD was widely discussed. Many children and young people expressed the psychological strain and negative emotions experienced when a parent was diagnosed with YOD and their attempts to cope with these feelings and experiences. There were three subthemes: poor psychological well‐being, grief and loss, and negative physical impact.

###### 3.2.1.1.1 Subtheme Poor Psychological Well‐Being

Children and young people discussed how having a parent with YOD resulted in a feeling of ‘emotional chaos’ (Johannessen et al. 2015), psychological strain (Allen et al. 2009), and a range of other negative emotions, including sadness, fear, anger, resentment, confusion, and frustration (Gelman and Rhames 2018; Hutchinson, Roberts, Daly, et al. 2016; Nichols et al. 2013; Sikes and Hall 2018b). Mental health conditions such as depression, anxiety, and psychosis (Hutchinson, Roberts, Kurrle, and Daly 2016) along with self‐harm (Gelman and Rhames 2018) were also experienced. In their mixed methods study, Svanberg et al. (2010) measured participants perceptions of burden using the Zarit Burden‐Short (Bédard et al. 2001) and found that over half (58%: *N* = 7) scored above the cut‐off for high burden. Perhaps adding to the perceptions of high burden, children reported the concealment of emotions to minimise or not to add to the family's heightened levels of stress, specifically to the parent without the diagnosis of YOD (Gelman and Rhames 2020; Hutchinson, Roberts, Daly, et al. 2016; Svanberg et al. 2010).

It is so hard: The consequence is that I am now left with anxiety, guilt, and unrest. I have so many ups and downs that I feel sick …. (Johannessen et al. 2015)

Children and young people experienced significant anxiety and uncertainty in their own future as their parent's dementia progressed, which adversely affected their psychological well‐being (Gelman and Greer 2011; Groennestad and Malmedal 2022; Millenaar et al. 2014; Sikes and Hall 2018a). They were also deeply concerned about the emotional strain and health of their parent without dementia (Allen et al. 2009; Aslett et al. 2019; Gelman and Greer 2011; Gelman and Rhames 2020; Lord 2009; Millenaar et al. 2014). Additionally, children and young people worried about their own health and well‐being and the impact their parent's diagnosis had on the future of their family (Gelman and Greer 2011; Hutchinson, Roberts, Kurrle, and Daly 2016; Lord 2009).

Children and young people adopted various methods to cope with their circumstances. While some used positive coping strategies, such as faith, immersion in academia, and extracurricular activities (Gelman and Rhames 2018) others reported negative coping strategies that adversely affected their wellbeing. These negative strategies included emotional detachment (Svanberg et al. 2010), substance misuse (Hutchinson, Roberts, Daly, et al. 2016), avoidance behaviour (Millenaar et al. 2014) and escapism (Johannessen et al. 2015).

I stabbed myself in the thigh with the fork, just because I didn't understand what was happening and I just was I guess just really gritting my teeth against freak out and depression and anxiety and dealing with the whole situation …. (John) (Hutchinson, Roberts, Daly, et al. 2016)

###### 3.2.1.1.2 Subtheme: Grief and Loss

Frequently expressed were feelings of grief and loss. Many experienced feelings of parental‐related losses, such as in their parent with YOD as now being a different person and of a sense of losing their ‘real’ parent, becoming a stranger and a sense of losing their previous, meaningful relationship (Aslett et al. 2019; Gelman and Greer 2011; Gelman and Rhames 2020; Johannessen et al. 2015). Alongside this, the progressive nature of dementia resulted in further feelings of grief including grief for who their parent used to be (Gelman and Rhames 2018), anticipatory grief (Allen et al. 2009; Groennestad and Malmedal 2022; Sikes and Hall 2018a), as well as feeling in a continuous state of grief (Lord 2009; Sikes and Hall 2018a).

So, you look at someone and you know who they are but … you're not quite sure… [. . .] but the way they're talking is like … completely different to how they would be. (Matthew) (Aslett et al. 2019)

###### 3.2.1.1.3 Subtheme: Negative Physical Impact

The impact of a parent being diagnosed with YOD on children's physical health was also expressed, including physical strain while addressing fall occurrences, a lack of sleep, weight fluctuation due to stress, and neglect of physical needs, such as access to food, personal care (Allen et al. 2009; Gelman and Greer 2011; Hutchinson, Roberts, Daly, et al. 2016).

… I had actually had enough of it and he hit me so I raised my hand back to him. I was actually going to hit him an' my mum had to get between us and I had to walkout. (P1) (Allen et al. 2009)

##### 3.2.1.2 Theme 2: Social Connection and Peer Support

Social isolation, withdrawal, and social exclusion were commonly expressed and emphasised the need for support from friends, peers, or others in a similar situation. This highlights the importance of maintaining social connectedness and support systems.

Social isolation and withdrawal from other children and young people, both within their families and peer groups was commonly reported (Gelman and Greer 2011; Hutchinson, Roberts, Daly, et al. 2016; Johannessen et al. 2015; Masterson‐Algar et al. 2023; Svanberg et al. 2010). Children often hid their parent's diagnosis from friends and peers (Millenaar et al. 2014), not inviting them to their house out of fear or embarrassment of their parent's behaviour (Allen et al. 2009; Gelman and Greer 2011; Lord 2009). Young people of working age also hid the parent's diagnosis from colleagues (Hutchinson, Roberts, Daly, et al. 2016).

Others do not understand how it feels to be a child and see your parent have these episodes of total confusion, or how he can get very angry, childish, and show little sympathy; they do not know that this is happening, they only think you become a bit forgetful. (P4) (Groennestad and Malmedal 2022)

Many children and young people felt socially excluded and both perceived and experienced stigmatisation and discrimination by the public, wider family and peers (Gelman and Rhames 2018; Hutchinson, Roberts, Daly, et al. 2016; Hutchinson, Roberts, Kurrle, and Daly 2016). They experienced changes to relationships with friends and family, with others distancing themselves or reducing their contact after a diagnosis was made (Allen et al. 2009; Groennestad and Malmedal 2022; Johannessen et al. 2015). Familial conflict with their parent without the diagnosis was also reported (Hutchinson, Roberts, Kurrle, and Daly 2016; Millenaar et al. 2014). Within schools, issues such as bullying (Sikes and Hall 2018b) and peers distancing themselves (Hutchinson, Roberts, Daly, et al. 2016) also contributed to their sense of social exclusion.

As a result of social exclusion, children and young people expressed a strong desire for emotional and peer support. Only one study measured resilience on a measure specific to an adolescent population (Svanberg et al. 2010), with participants showing moderate levels of resilience and acknowledging the value of peer support in developing and maintaining resilience; however, the sample size was small (*N* = 12). Consequently, support groups (Hutchinson, Roberts, Daly, et al. 2016; Johannessen et al. 2016) and peer contact (Millenaar et al. 2014; Nichols et al. 2013) were highly valued.

##### 3.2.1.3 Theme 3: Preserving Childhood and Adolescence

When a parent was diagnosed with YOD, it disrupted the lives of children and young people, emphasising the need to support their development and well‐being during childhood and adolescence. In cases where role reversal occurs, children experienced parentification, taking on increased responsibilities and having to mature quickly (Johannessen et al. 2015; Svanberg et al. 2010). Children might assume the role of a second parent, becoming caregivers who provide physical and medical care, as well as emotional, practical, and financial support (Gelman and Rhames 2018, 2020; Groennestad and Malmedal 2022; Lord 2009; Millenaar et al. 2014). Balancing multiple roles and duties, such as being a carer, student (Nichols et al. 2013; Sikes and Hall 2018b), and engaging in paid work (Hutchinson, Roberts, Kurrle, and Daly 2016) further disrupts the expected childhood experience.

Many children felt personally responsible for keeping the family together (Groennestad and Malmedal 2022; Hutchinson, Roberts, Daly, et al. 2016) as they felt an increased dependence on them from the parent without the diagnosis of dementia, including a perceived need for companionship and conversation (Gelman and Greer 2011) and to act as a confidante and source of support for other family members to help them cope with the impact of YOD (Aslett et al. 2019; Nichols et al. 2013).

Interruptions to childhood and adolescence can also impact educational aspirations and plans, such as opting for local universities or colleges to study closer to home, delaying school, leaving university, enrolling in distance learning, or even declining scholarships and university offers altogether (Gelman and Rhames 2020; Groennestad and Malmedal 2022; Hutchinson, Roberts, Kurrle, and Daly 2016; Sikes and Hall 2018b). There were other reported changes to life plans, including postponing moving out of the family home (Millenaar et al. 2014) and changing career paths to be on hand to support the care of the parent with YOD (Allen et al. 2009). The experience of individual losses such as opportunities, childhood (Hutchinson, Roberts, Kurrle, and Daly 2016; Lord 2009) and a future (Gelman and Rhames 2020), further highlights the need to maintain childhood and adolescence for children and young people affected by YOD.

Since I was 15, when I knew that dad wasn't going to be bringing in any money for mum, it kind of put me more towards work than university and college. Like my sister and brother, both went to Uni and mum, well dad supported them both and as I say I went into (. . .) which is a trade that I can use apart from being at work. (P11) (Allen et al. 2009)

##### 3.2.1.4 Theme 4: Practical Support

Children and young people's need for practical support was evident in their desire for their needs to be recognised and in receiving assistance from formal services. Two subthemes emerged, including practical support that embraced support services and needs, and the role of education.

###### 3.2.1.4.1 Subtheme: Support Services and Needs

The lack of informal and formal support available for children and young people affected by YOD was often referred to (Gelman and Rhames 2018; Hutchinson, Roberts, Daly, et al. 2016). These include a lack of familial support (Hutchinson, Roberts, Kurrle, and Daly 2016), information and resources aimed at a child or young person (Groennestad and Malmedal 2022; Lord 2009) and poor public services specific to children and young people (Johannessen et al. 2015). The desired practical and tangible support needs identified involve a family approach which is inclusive of children and young people (Svanberg et al. 2010) and timely and targeted information for children and young people across the trajectory of YOD, from diagnosis onwards (Millenaar et al. 2014).

There was also a misunderstanding as to who would refer the young person. So, education presumed social work would be referring and social work presumed education staff would be referring. And it turned out no one was referring the young person, so they were just kind of left dangling, which is really sad. (P1) (Masterson‐Algar et al. 2023)

Health and care professionals and parents pointed out the need for children and young people to be formally identified as a carer and included in support services that are family centred (Masterson‐Algar et al. 2023). When care was provided to the parent with YOD in their own home (Hutchinson, Roberts, Kurrle, and Daly 2016) and children and young people were able to get ‘distance’ from directly providing care (Groennestad and Malmedal 2022), they expressed feeling relief, further highlighting the need for practical support.

###### 3.2.1.4.2 Subtheme: Role of Education

Education was seen as providing a source of stability (Hutchinson, Roberts, Kurrle, and Daly 2016) and a distraction (Sikes and Hall 2018b) for children and young people. However, the experiences of support received by children and young people varied across educational levels and institutions. At primary level, emotional literacy, art therapy and flexible time off were available to support some children of a parent with YOD (Hutchinson, Roberts, Daly, et al. 2016; Sikes and Hall 2018a). However, at secondary level, students felt there was a lack of understanding around YOD among staff (Sikes and Hall 2018b). Whilst many felt initially supported at university, this support soon faded and lacked any direction or continuity (Sikes and Hall 2018b). Educational institutions have the potential to be sources of support for children and young people living with a parent diagnosed with YOD at this important developmental stage of their lives.

My school didn't really understand what I was going through, no one was there to help me through it… the teachers weren't there, there wasn't a counsellor… My dad passed away on the Wednesday and I had a GCSE exam… on the Friday and so I went in to do the exam because I didn't like to give up on stuff… I don't like people taking pity on me, don't like that attention, didn't really want that at that age and I overheard a teacher say ‘oh yeah she's in for this one exam but then delay it’ and that was talking about me in front of a load of other people, not very respectful really. (Frankie, 23) (Sikes and Hall 2018b)

## 4 Discussion

The findings of this review of international literature provide an important and contemporary summary of the evidence of the needs of children and young people of a parent diagnosed with YOD and present a clear focus on the specific needs of this underserved population from which services of any country could respond. Many children will experience pressures, issues, and needs common to adult carers of a person with dementia, for example, anticipatory grief and loss (Blandin and Pepin 2017) and require information and education about dementia (Mansfield et al. 2023). However, children and young people of a parent diagnosed with YOD also have unique needs and requirements that are specific to their age and developmental stage and are crucial for their psychological health and well‐being. These needs include support in developing and maintaining relationships with their peers, managing embarrassment and stigma in an educational context, and maintaining their educational focus and early life plan. Although these issues have been identified in research for over 15 years (2009 to present day), children and young people's needs are often neglected in international and national dementia strategies, policy, and, importantly, in dementia services provision.

The negative impact of the unmet emotional and psychological needs of children and young people of a parent with YOD is cause for concern internationally; however, we have found little evidence to suggest that, given the international literature in this review, few countries have responded with a service offer. In the UK, as in other countries, such a state of affairs may also contribute to the rising prevalence of mental problems in children and young people in general (World Health Organistaion (WHO) 2024; UNICEF 2024; NHSE 2023). When considering a service response from a single country, there are many issues that can affect the mental health and well‐being of children and young people, such as bullying, substance use, self‐harm, and concerns about cost of living, education, climate change, and the future that may be compounded and exacerbated for those experiencing a parent with YOD. In a recent UK NHS survey, one in five children and young people (8–25 years) experienced a mental health disorder in 2023 (NHSE 2023). Mental Health Services reported having supported over 700,000 children and young people in 2023, a 47% increase on previous years. However, of these, less than half received the specialist care of NHS Child and Adolescent Mental Health Services (CAMHS) (NHSE 2023). Children and young people whose parents have a serious physical and/or mental illness are at higher risk of experiencing anxiety, depression, somatic complaints, and social withdrawal (De Roos et al. 2017, 2022; Dam and Hall 2016). There are many similarities in these factors when considering children and young people with a parent diagnosed with YOD (Poole and Patterson 2020).

The physical impact on the health of an adult carer of a person with dementia has been widely researched, often showing that carers can incur high levels of morbidity compared to carers of other conditions (Alzheimer's and Dementia 2024). This review found some evidence suggesting that the physical health of children and young people might be impacted by providing care. The potential for physical neglect, sleep disturbances, and back pain from caring duties associated with moving and handling and/or responding to falls of the parent with YOD exists. This area has not been extensively studied, and results of the included studies may not individually be considered generalisable; however, collectively they do suggest needs in this population that not only warrant the development of a service response but also further research, particularly to understand any long‐term consequences. Physical health challenges are also likely to be compounded by the stress and emotional strain of their caring role. Similarly, research suggests that young carers generally are 1.5 times more likely than their peers to have a disability or a special educational need (Scottish Government 2017) is of equal concern for this population.

Similarly, children and young people experience significant impacts on their relationships, both within the family and with their peers. A large body of research exists on the impact of dementia on the relationships with adult carers, for example, spouses (Macdonald et al. 2020; Cui et al. 2024; Van Hout et al. 2025) and many services designed to support them in maintaining relationships. Children and young people also need supportive relationships both within and outside their family to help reduce experiences of isolation and stigma. Providing this support requires interventions that span across the home and the educational system, ensuring that support teachers and educators understand the impact of a parent's diagnosis of YOD and the needs of their children. The impact on education and future planning for children and young people raises important questions about educational attainment, school attendance, and future career prospects, with research needed at a national level to follow up the impact on young people over time.

### 4.1 Influencers for Bespoke Service Development

We know that service provision for families affected by YOD are lacking (Stamou et al. 2022). In ‘The Angela Project’, Stamou et al. (2022) successfully identified core features to inform service design for people with YOD using appreciative inquiry. However, the development of services specific to certain populations is often influenced by the numbers of people affected. We do not know with certainty the numbers of children and young people who are affected by a parental diagnosis of YOD, which may be an inhibiting factor for an appropriate national service response. This being said, Hassan et al. (2024) analysed data from a cross‐sectional study of 1371 adolescents' (11–18 years) experiences of dementia and found that 64% had encountered a person with dementia and 25% had helped to look after a person with dementia. However, the study provides no data to indicate the numbers of adolescents who lived with and or cared for a parent with YOD. Similarly, there remains a gap in hearing the voices and experiences of children below the age of 11 years, as this data was not available in many of the studies reviewed. Having a greater understanding of the numbers and age ranges of children affected by a parental diagnosis of YOD and needs specific to their age may present a more compelling case for bespoke service provision specific to this population.

### 4.2 Developing an Admiral Nurse Model for Children and Young People

Meeting these needs requires specialist skills and knowledge, both about the nature and progression of YOD but also in understanding the psychological and mental health needs of the child or young person, alongside consistency in a bespoke approach to adapt to their changing needs. In a scoping review, Grundberg et al. (2021) reviewed what is known from the experiences and perspective of a child or young person where a parent was diagnosed with YOD. Whilst our review concurred with many of their findings, in addition we identified the key relational elements impacted that would constitute a bespoke intervention to support this population, from both a psychological and social level, also including an educational perspective. In response to these needs and the findings of the literature review, the charity Dementia UK has developed a Consultant Admiral Nurse post specifically to support children and young people who have parents with YOD. The functions of this innovative role have been mapped against the early evaluation data from the service and the findings of this systematic review (Table 3).

**TABLE 3:** Identified needs of children and young people matched to role of Admiral Nurse.

Domains identified in the literature	Need(s)	Role of Consultant Admiral Nurse for Children and Young People
Knowledge and information	Information about YOD that is tailored to age and level of understanding of the child/young person Delivering and adapting information as the child/young person ages and develops	Specialist knowledge and clinical experience supporting families with YOD and rarer dementias Work in partnership with the parent or guardian Provide bespoke support to improve a child or young person's comprehension and understanding of YOD and type diagnosed and provide age‐appropriate support/education Explore child or young person's observations and perceptions of changes dementia has on parent and provide education to explain such changes and effect on different areas of the brain, such as, memory, language, behaviour, sight, personality, etc Provide advice on ‘what is a carer’ and how this new role impacts family dynamics and routines Uses advanced skills in the assessment, treatment and case work of children and young people living with the impact of YOD Employ a range of evidence‐based psychosocial interventions pertinent to supporting children and young people Support families in complex decision making in relation to care, risk identification and management to all family members affected by YOD Detailed knowledge and practice within the nursing legal and ethical frameworks, Mental Capacity Act, Mental Health Act and Safeguarding procedures
Emotional support	Support to understand the changes taking place within the family due to a parent's YOD Support in understanding their own needs and feelings Supporting the child and young person to balance demands made upon them as ‘carers’ with their own needs as a child/young person	Develop a trusted relationship with child or young person Deliver specialist advice, support and therapeutic interventions for children and young people living in families facing YOD Use active listening so providing a safe place for a child or young person to both identify, share and validate their emotions Explore suitable and evidence based coping strategies Provide information about pastoral support available within education settings and empower a child or young person to access Share information about national mental health charities and organisations aimed at supporting children and young people Identify any needs for the involvement of other specialist support services, such as, counselling, CBT, GP Support to manage and diffuse distressing and emotional situations for children and young people and their families, often at times when they have difficulty communicating or understanding
Maintaining consistency in the child's own educational and developmental growth	Support both the child/young person and parents in maintaining stability in a child/young person's developmental and educational pathway by: – Avoidance of withdrawing from previously enjoyed educational and school related activities – Recognising when there is a risk of the child/young person negating their own needs – Avoid changing long held career and educational aspirations – Liaising between parents, child/young person and educational institution to ensure developmental needs are met	Skilled in identifying wellbeing and education needs to empower children and young people to focus on their own wellbeing, hobbies and activities Actively promote the importance of child or young person's own future planning and educational/career aspirations and prospects Empower families to access education pastoral supports Provide education and resources on dementia for teachers and school staff. Deliver specialist advice on sharing how a diagnosis of YOD can impact education, learning, homework, exams, revision and concentration Identify and manage complex safeguarding incidents and escalate to local safeguarding teams NSPCC Level 4 Safeguarding Children Trained Develop bespoke resources for children and young people on aspects of living with a parent with a diagnosis of YOD
Maintaining social connections	Support to maintain connection with peers and groups Support with feelings of stigma and embarrassment of their parent with YOD Support the child/young person and other service providers, such as schools, in instances of social exclusion, bullying and harassment Encourage hobbies and activities to support wellbeing	Provide expert advice, education and support in addressing stigma and lack of awareness of YOD Provide support on strategies for sharing a parent's diagnosis with peers, teachers, and other family members Signpost to young carer support and young carers assessment Provide safe space and actively listen to concerns about being embarrassed about the parent with YOD Support child and young person to maintain relationships with parent diagnosed with YOD through, for example, life story work, music and other activities to support positive engagement Explore ways to raise awareness of dementia locally, such as sharing resources, local awareness events
Maintaining psychological well‐being	Support child/young person to understand and manage any negative emotions (sadness, fear, anger, resentment, confusion and frustration) which place them at risk of developing mental health conditions Support the development of positive coping strategies and minimise concealment of emotions Support an awareness of negative coping strategies (dependent upon and in line with the child/young person's age)	Demonstrate the ability to provide highly complex or sensitive information, where developed persuasive, motivational, negotiating, empathetic and/or reassurance skills are required Develop confidence in a child or young person to share their emotions, negative experiences and troubles through providing a trusted adult relationship. Actively listening and validating their emotions in a non‐judgemental way Promote good personal mental health awareness in a child or young person Explore with and employ evidence based coping strategies and interventions tailored to the child or young person Identify need for escalation to specialist support services, such as counselling, CBT, GP, pastoral support
Maintaining physical well‐being	Support an awareness of personal physical health and well‐being	Deliver advise, support and therapeutic interventions for children and young to promote physical health and wellbeing, such as: – Importance of access to outdoor space – Maintaining and developing hobbies – Good sleep hygiene – Healthy diet – Signposting and supporting access to local, support groups – Prioritising self‐care
Recognising and working with grief and loss	Support the child/young person's understanding of grief and anticipatory grief, in particular when: – The health of parent with YOD deteriorates – Parent with YOD seems to be a different person – Loss/changes to the relationship with parent with YOD – There are changes to family life and structures – There are changes and losses to childhood experience	Provide education and support for children and young people in understanding feelings of grief and loss Identify the need for other specialist support, such as counselling Work in partnership with well parent or guardian Provide age‐appropriate information about the stages and progression of dementia Identify when isolation from peers and loss of social contacts is an issue Empower child/young person to engage with education pastoral support

*Note:* Many of the skills and attributes of the Consultant Admiral Nurse for Children and Young People are employed across all domains of need but are detailed here in an illustrative manner to avoid duplication.

Evidence shows that for adult carers of a person with dementia, feeling supported by the ongoing presence of a specialist in dementia who knows them and their needs well engenders confidence to cope in spite of the difficulties and uncertainties they face (Gridley et al. 2019). This specialist nursing model for children and young people will be evaluated to understand if a specialist nursing intervention, bespoke to this population, can be equally impactful.

### 4.3 Strengths and Limitations

A strength of this study is that it combines a review of the literature of the needs of children and young adults where a parent is diagnosed with young onset dementia and practically aligns these to a bespoke service response of a nursing intervention.

This study also has limitations. Firstly, though the search strategy, including the selection of databases and search terms, was guided in consultation with an academic librarian, the inclusion of other databases, such as EMBASE, may have added to the yield of relevant studies. Secondly, whilst we found that the MeSH terms used were sufficient to retrieve a comprehensive and relevant set of studies aligned with our inclusion criteria within the chosen databases, it is worth noting that these terms may have identified additional studies in other databases, such as EMBASE.

## 5 Conclusion

This review highlights the significant and overlooked needs of children and young people who have a parent diagnosed with YOD. While many of their challenges align with those faced by adult carers, children and young people also experience unique difficulties related to their developmental stage, social relationships, and educational pathways. Despite a growing awareness of the mental health crisis among young people, the specific needs of this group remain absent from national dementia strategies, policy frameworks, and service provision.

The lack of precise data on the number of affected children and young people may be a barrier to countries developing appropriate national support services, reinforcing the need for dedicated research in this area. To address these gaps, tailored interventions that include home, education, and healthcare settings are essential. The development of a Consultant Admiral Nurse role to support this specific population represents an important step in creating specialised support for this population. However, a broader, systemic response is needed to ensure that children and young people receive the recognition and assistance required to safeguard their well‐being and future opportunities.

## Author Contributions

All authors have agreed on the final version and meet at least one of the following criteria (recommended by the ICMJE*): (1) substantial contributions to conception and design, acquisition of data, or analysis and interpretation of data; (2) drafting the article or revising it critically for important intellectual content. T.S., E.W., P.J., M.W., A.P. and K.H.D. made substantial contributions to conception and design, or acquisition of data, or analysis and interpretation of data. T.S., E.W. and K.H.D. involved in drafting the manuscript or revising it critically for important intellectual content. K.H.D., T.S., E.W., P.J., M.W., A.P. and H.G. given final approval of the version to be published. Each author should have participated sufficiently in the work to take public responsibility for appropriate portions of the content. T.S., E.W., K.H.D. agreed to be accountable for all aspects of the work in ensuring that questions related to the accuracy or integrity of any part of the work are appropriately investigated and resolved.

## Ethics Statement

The authors have nothing to report.

## Consent

The authors have nothing to report.

## Conflicts of Interest

The authors declare no conflicts of interest.

## Supporting information


**File S1:** jan70205‐sup‐0001‐FileS1.xlsx.

## Data Availability

Data available on request from the authors.
